# *Ixeris dentata* and *Lactobacillus gasseri* Extracts Improve Salivary Secretion Capability in Diabetes-Associated Dry Mouth Rat Model

**DOI:** 10.3390/nu12051331

**Published:** 2020-05-07

**Authors:** Hwa-Young Lee, Mingkun Gu, Jinhua Cheng, Joo-Won Suh, Han-Jung Chae

**Affiliations:** 1 Department of Pharmacology and Institute of New Drug Development, Jeonbuk National University Medical School, Jeonju, Jeonbuk 561-180, Korea; youngat84@gmail.com; 2 Interdisciplinary Program of Biomodulation, Myongji University, Yongin, Gyeonggi 17058, Korea; 19930409gmk@gmail.com; 3 Center for Nutraceutical and Pharmaceutical Materials, Myongji University, Yongin, Gyeonggi 17058, Korea; jhcheng316@mju.ac.kr (J.C.); jwsuh@mju.ac.kr (J.-W.S.)

**Keywords:** *Ixeris dentate*, *lactobacillus*, salivation, xerostomia

## Abstract

Dry mouth, hyposalivation, or xerostomia is a significant problem in diabetic patients; however, there has been no way to relieve these symptoms. This study’s aim was to evaluate the effects of *Ixeris dentata* (IXD) in combination with lactobacillus extract on the salivation rate in diabetes-induced dry mouth, and its mechanism was also investigated. In the streptozotocin (STZ)-induced diabetes model, the dry mouth condition was established as a model. Here, rats were treated with water or IXD through the sublingual spray, and subsequently treated with or without a spray of lactobacillus extract. In diabetes condition, the salivary flow rate, amylase activity, and aquaporin-5 and Na^+^/H^+^ exchanger (NHE-1) expressions were markedly decreased, whereas they were more significantly recovered in the sequential treatment of IXD-lactobacillus extract than in each single treatment. Furthermore, oxidative stress and its related ER stress response were especially regulated in the IXD/lactobacillus extract condition, where the following anti-oxidative enzymes, glutathione assay (GSH: GSSG) ratio, superoxide dismutase (SOD), and glutathione peroxidase (GPx), were involved. This study suggests that the combination of IXD and lactobacillus would be a potential alternative medicine against diabetes-induced hyposalivation and xerostomia.

## 1. Introduction

Diabetes mellitus is a kind of metabolic disorder where high blood sugar level persists over a long period and has a physical consequence on patients. Xerostomia (dry mouth), which causes difficulties in swallowing, chewing, and increased risk of oral problems, is one of these effects [1]. Over the years, several works have investigated the prevalence of oral lesions and xerostomia in diabetic patients. Previous studies reveal that 76.4% of diabetic patients suffer from xerostomia [1], which has affected their quality of life. Hence, dry mouth needs to be diagnosed and treated to improve oral health and better quality of life [2].

IXD is one of the herbal medicines routinely used in Korea, Japan, and China to reduce the treatment of diabetes, indigestion, and allergies [3,4]. Additionally, in these countries, IXD extract is widely popular as a functional or healthy food [5]. In our previous efforts, *Ixeris dentata* (IXD) was identified as a regulator of salivary secretion using a diabetic rat model [6]. Additionally, several studies have reported the nutritional value of IXD and its components [5]. Antioxidant effects of IXD have been confirmed similar to flavonoid-enriched natural extracts. However, its specific functions in a diabetes-induced dry mouth model need to be investigated.

It has been reported that oxidative stress plays a role in reducing the saliva secretion [7], and oxidative stress is influenced by reactive oxygen species (ROS), which also affect age-related diseases, including diabetes, obesity, and hyperlipidemia [8]. Metabolic diseases increase the mitochondrial production of ROS, decreasing the antioxidative potential of the body [9]. As a consequence, a high degree of challenge is presented to the body to maintain the redox balance, ultimately accumulating ROS. Furthermore, accumulated oxidative stress is deleterious to cell membrane proteins and phospholipids and leads to cellular dysfunction [10]. Dysmetabolism-associated saliva dysfunction has been reported to be related to redox imbalance and ROS accumulation [11].

Moreover, the use of lactic acid bacteria is popular in fermented foods around the world and is well-accepted by society. Also, a few strains of lactic acid bacteria are routinely used in probiotics for their health benefits. Lately, several reports suggesting the beneficial effects of lactic acid bacteria, such as immunoregulatory, antioxidative, and anti-inflammatory effects, have been reported [12,13,14], representing the safe and valuable functional food ingredients. Some strains also contribute to the maintenance of oral hygiene and salivary secretion function [15]. The lactic acid bacteria strains combined or co-treated with the defined salivary secretion enhanced material “IXD” can be considered to show a dual function or synergistic effect in saliva function maintenance. Further, to document the possible synergistic or additive effect with combined or co-treated materials, scientific evidence needs to be established.

Considering the natural health benefits of IXD and lactic acid bacteria, the effects of co-treated IXD and lactobacillus extract were investigated to improve the dry mouth condition in a diabetes-associated dry mouth model. The utilization of IXD and lactobacillus extract may indicate the potential activity of the co-treated materials against the hyposalivation and its related redox disturbance mechanisms.

## 2. Materials and Methods

### 2.1. Chemicals and Reagents

Pilocarpine hydrochloride, streptozotocin (STZ), and citric acid were procured from Sigma Chemical Company (St. Louis, MO, USA). The following proteins were used in this study: antibodies against anti-amylase (#4017, Cell Signaling Technology, Danvers, MA, USA), anti-NHE-1 (sc-28758, Santa Cruz Biotechnology, Inc., Santa Cruz, CA, USA), anti-AQP-5 (sc-514022, Santa Cruz Biotechnology, CA, USA), anti-GRP78 (sc-376768, Santa Cruz Biotechnology, CA, USA), anti-CHOP (#2895, Cell Signaling Technology, Danvers, MA, USA), p-IRE1α (ab48187, Abcam, Cambridge, MA, USA), IRE-1α (#3294, Cell Signaling Technology, Danvers, MA, USA), anti-p-eIF2α (#9721, cell signaling, Danvers, MA, USA), anti-eIF2α (sc-133132, Santa Cruz Biotechnology, CA, USA), and anti-β-actin (sc-130300, Santa Cruz Biotechnology, CA, USA). Horseradish peroxidase-conjugated secondary antibodies were obtained from Enzo Life Sciences, Inc. (Farmingdale, NY, USA).

### 2.2. Plant Material Preparation

The National Institute of Horticultural and Herbal Science (NIHHS), Rural Development Administration (RDA), Wanju, Korea, confirmed the identification of Ixeris dentata roots harvested in 2014 at Dangin, Korea (ID 2014-01). Later, it was deposited at the College of Pharmacy, Yonsei University, Incheon, Korea [6]. Roots were dried and powdered, and about 40 g of powdered root was extracted with 300 mL of water and ethanol in a gradient manner (20%, 40%, 60%, 80%, and 100% ethanol) using an ultrasonic apparatus for 3 h at 50 °C. These extracts were suspended in water to get the desired concentration before use.

### 2.3. Preparation of Lactobacillus Extracts

*Lactobacillus gasseri* MJM6064 was isolated from human saliva and stored in 20% glycerol at −80 °C. It was activated on a DeMan-Rogosa-Sharpe (MRS) agar plate at 37 °C for 24 h. The cells were precultured in MRS broth at 37 °C for 16 h. Further, 500 µL of preculture was inoculated into 0.5 L of MRS broth and incubated at 37 °C for 24 h. After fermentation, the supernatant was extracted with the same volume of ethyl acetate (EtOAc), and the extract was concentrated to dryness by a rotary evaporator under vacuum. The dryness was suspended in water for further use.

### 2.4. Induction of Diabetes

Type 1 diabetes was induced as described previously [16]. Briefly, 65 mg/kg of STZ was dissolved in a 0.1 M citrate buffer. The citrate buffer was used as a vehicle, and after three days of injection, basal blood glucose levels were measured in overnight-fasted rats. A glucometer (Accu-Chek, Roche, Germany) was used to assess the systemic glucose concentration. Blood glucose concentrations of 300 mg/dL or higher are regarded as diabetic and these diabetic animals were used for the study [17].

### 2.5. Experimental Design

Sprague-Dawley male rats (250–270 g) were procured from the Samtako (Daejeon, Republic of Korea) and maintained in specific pathogen-free housing condition at 22 ± 2 °C and 55 ± 5% humidity under 12 h light/dark cycle. Rats were cared for in accordance with the regulations of the Institutional Animal Care and Use Committee of Jeonbuk National University Laboratory Animal Center (cuh-IACUC-2018-2). A week earlier, all the animals used in the study were acclimatized to experimental conditions. Rats were randomly separated into 8 groups, consisting of 10 rats in each group. Briefly, two weeks after either water or STZ injection, the rats were anesthetized prior to sublingual treatment (1 spray = 50 µL). Different groups of the study are as follows: the normal control group was sprayed with water (control + water), the second normal group was sprayed with IXD extract (control + IXD, 10 mg/kg), the third normal group was sprayed with lactobacillus extract (control + *Lactobacillus gasseri*, 0.5 mg/kg), and the fourth normal group was sprayed with IXD and cotreated lactobacillus extract (control + IXD/*Lactobacillus gasseri*). Similarly, diabetic rats were grouped as control rats sprayed with water (STZ + water), rats sprayed with IXD extract (STZ + IXD, 10 mg/kg), rats sprayed with lactobacillus extract (STZ + *Lactobacillus gasseri*, 0.5 mg/kg), and rats sprayed with IXD and cotreated lactobacillus extract (STZ + IXD/*Lactobacillus gasseri*). Post saliva collection, the animals were sacrificed to collect the submandibular glands. Excised submandibular glands were weighed carefully; a section of the lobe was used for histological examinations, and another was immediately frozen using liquid nitrogen (Supplementary Figure S1).

### 2.6. Collection of Total Saliva

Saliva was collected as described previously [18]. Briefly, two weeks after STZ or vehicle injection, rats were anesthetized prior to sublingual treatment with water or IXD extract (10 mg/kg). Later, 0.6 mg/kg of pilocarpine is injected intraperitoneally, and saliva was gathered utilizing pre-gauged, cotton balls for up to 30 min [6].

### 2.7. Immunoblotting

Immunoblotting was performed as described previously [6] using submandibular gland tissue homogenates and saliva. Briefly, submandibular glands were homogenized in a radioimmunoprecipitation assay lysis buffer and were quantified for total protein using a Bio Rad Protein assay buffer (Bio-Rad, Hercules, CA, USA). About 30 µg of total protein extract was separated on SDS-polyacrylamide gel electrophoresis (SDS-PAGE; BioRad, Hercules, CA, USA) and transferred to a polyvinylidene fluoride membrane (PVDF). Later, membranes were probed with indicated primary antibodies and incubated again with the secondary antibody. Post incubation blots were developed using a chemiluminescence detection system. For Coomassie Brilliant Blue R-250 staining (CBB), samples were stained with CBB and de-stained using a 30% methanol/10% acetic acid solution.

### 2.8. Hematoxylin and Eosin Staining

Hematoxylin and eosin (H&E) staining was performed as described previously [6]. Briefly, processed paraffin-embedded submandibular gland tissue was sectioned, deparaffinized, rehydrated, and washed with water. Later, it was stained with H&E and dehydrated and observed under a microscope.

### 2.9. Immunohistochemistry

Immunohistochemistry was performed as described previously [6]. Formalin-fixed, paraffin-embedded salivary gland tissues were deparaffinized and rehydrated in xylene, followed by decreasing the ethanol concentration. Antigen retrieval was accomplished using a Target Retrieval Solution in a decloaking chamber (Biocare Medical, Concord, CA, USA). Further, sections were cooled, rinsed, and blocked with a phosphate buffer containing hydrogen peroxide and detergent to block endogenous peroxidase activity. Prior to incubation with a primary antibody, sections were washed with Tris-Buffered Saline-Tween 20 (TBST). Later, the sections were rinsed and treated with secondary antibodies at room temperature. Then, sections were washed and incubated with 3-amino-9-ethylcarbazole (AEC) substrate chromogen before counterstaining with Harris hematoxylin (Sigma-Aldrich Co., St Louis, MO, USA). All the sections were washed thoroughly and mounted in an aqueous medium.

### 2.10. Dihydroethidine (DHE) Staining

DHE staining was done as described previously [19]. DHE treated tissue sections were incubated in a humidified chamber at 37 °C for 30 min. Fluorescence was detected with a fluorescent microscope (Olympus, Melville, NY, USA).

### 2.11. Statistical Analysis

All observations in this study are presented as mean ± SEM. Mean and standard deviations of the analyzed samples were determined, and one-way analysis of variance (ANOVA) was used to compare the groups, followed by a t-test. A *p*-value of <0.05 was considered statistically significant.

## 3. Results

### 3.1. IXD and Cotreated Lactobacillus Extract Shows Increasing Effect against Dry Mouth

**Figure 1:**
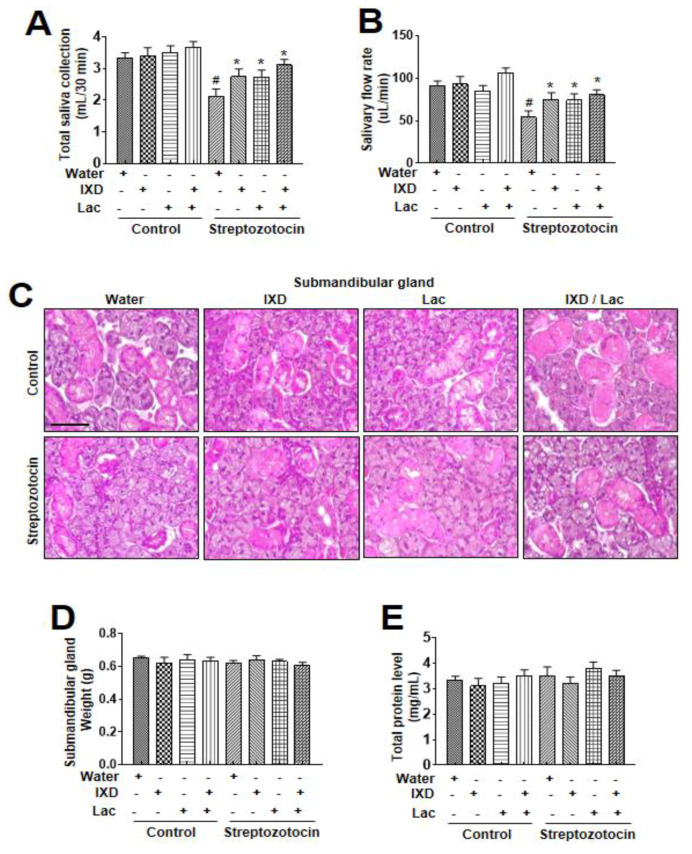
*Ixeris dentata* (IXD) and lactobacillus extracts improve salivary function in diabetes model. Water, IXD, or lactobacillus extract was given as a spray or IXD, and subsequently, lactobacillus was given as a spray to STZ-induced diabetes models. Saliva and the submandibular glands were collected after sacrificing the animals. Total saliva collected in 30 min (**A**) and salivary flow rate (**B**) were measured. (**C**) Hematoxylin and eosin staining were performed on paraffin-embedded submandibular gland tissues from normal and diabetic rats that were either treated with or without water, IXD, lactobacillus extract, or cotreated IXD and lactobacillus extracts. Magnification = 20×, scale bar = 100 μm. Weights of submandibular gland (**D**) and total salivary protein concentration (**E**) were measured. ^#^ significant difference vs. water-treated control rats; * significant difference vs. STZ-induced diabetic control rats (*p* < 0.05). Values are represented as mean ± SEM (*n* = 10 rats per group). STZ: streptozotocin.

Dry mouth is associated with decreased salivary secretion, and decreases in salivary secretion are reported to be closely associated with the diabetic condition [20,21]. These close associations between salivary secretion, dry mouth, and diabetic condition lead the current investigation where the influence of IXD, lactobacillus extract, and co-treatment was investigated in diabetes-induced dry mouth condition. A single spray of IXD, lactobacillus extracts, or the cotreatment was administered sublingually to normal or diabetic rats to observe the locally-acting effects of the drug. There was no significant difference in the saliva collection and salivary flow rate among water, IXD, lactobacillus, or cotreated control groups. However, IXD, lactobacillus, or its cotreatment recovered the saliva collection in streptozotocin-induced diabetes rats in which saliva output was markedly decreased (Figure 1A). Among the three conditions, the co-treatment condition showed a more significant increase in saliva collection and saliva flow rates than each single treatment condition (Figure 1A, Figure 1B). Further, to investigate the effects of IXD and lactobacillus extract on the morphology of the submandibular glands, histological examination with H&E was performed. The STZ-induced diabetic group showed depleted acinar cells and irregular ductal cell morphology in the submandibular glands (Figure 1C). Both the sublingual route and spray form of the IXD treatment may have enhanced salivation without improving the morphology of the salivary gland. We found no significant differences in submandibular glands weight in STZ-diabetic rats or IXD and cotreated lactobacillus extract (Figure 1D). The total protein concentration remained unchanged in all the groups (Figure 1E), suggesting that IXD/lactobacillus extract increase saliva secretion not due to the change of salivary gland weight.

### 3.2. IXD and Cotreated Lactobacillus Extract Increase Salivary Amylase Expression in Diabetic Rats

**Figure 2:**
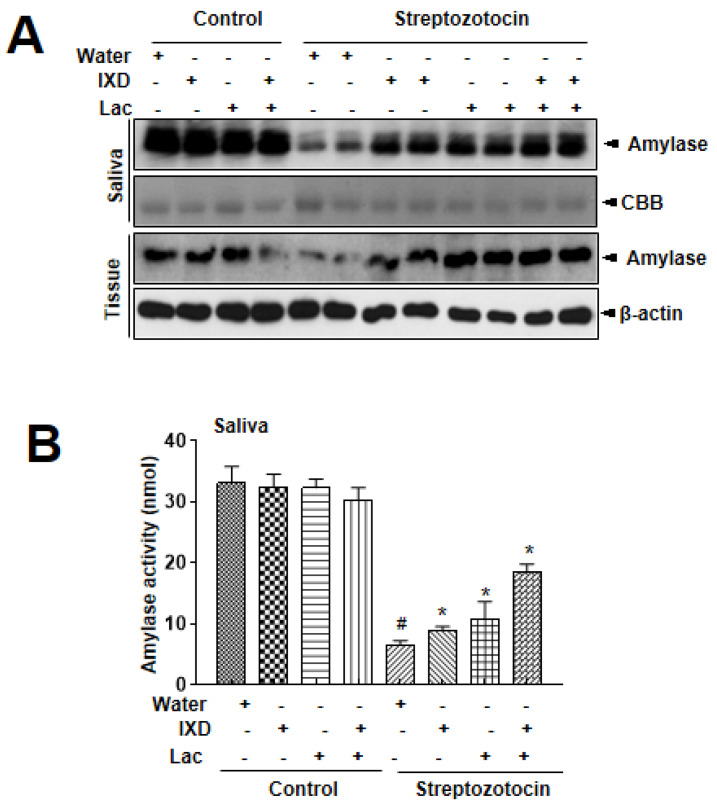
*Ixeris dentata* and lactobacillus extracts increase α-amylase expression in salivary glands from diabetic rats. Water, IXD, or lactobacillus extract were given as a spray or IXD, and subsequently, lactobacillus was given as a spray to STZ-induced diabetes models. Western blotting was performed with an anti-amylase antibody in submandibular gland tissue homogenates (**A**) and amylase activity was analyzed as described in Materials and Methods (**B**). β-actin was used as a loading control. ^#^ significant difference vs. water-treated control rats; *significant difference vs. STZ-induced diabetic control rats (*p* < 0.05). Values are represented as mean ± SEM (*n* = 10 rats per group). STZ: streptozotocin.

In addition to saliva secretion, the expression of α-amylase in saliva and salivary gland lysates represents an oral functional state [22]. In immunoblot analysis, no significant differences were observed in control rats treated with either water, IXD, lactobacillus extract, or IXD/lactobacillus extract (Figure 2A). However, significant reduction in amylase expression in both saliva and submandibular glands tissue were observed in STZ-induced diabetic rats. Interestingly, the expression of amylase was more significantly increased in both saliva and tissue lysates under the single spray of IXD, lactobacillus extracts, or cotreatment (Figure 2A). Amylase activity was also recovered in the presence of IXD combined with lactobacillus extract (Figure 2B), suggesting that cotreatment with IXD/lactobacillus extract restores the decreased amylase folding and the secretion seen in the salivary glands of the diabetic condition.

### 3.3. IXD and Cotreated Lactobacillus Extract Increase Salivary Secretion Through the Activation of AQP5 and NHE-1

**Figure 3:**
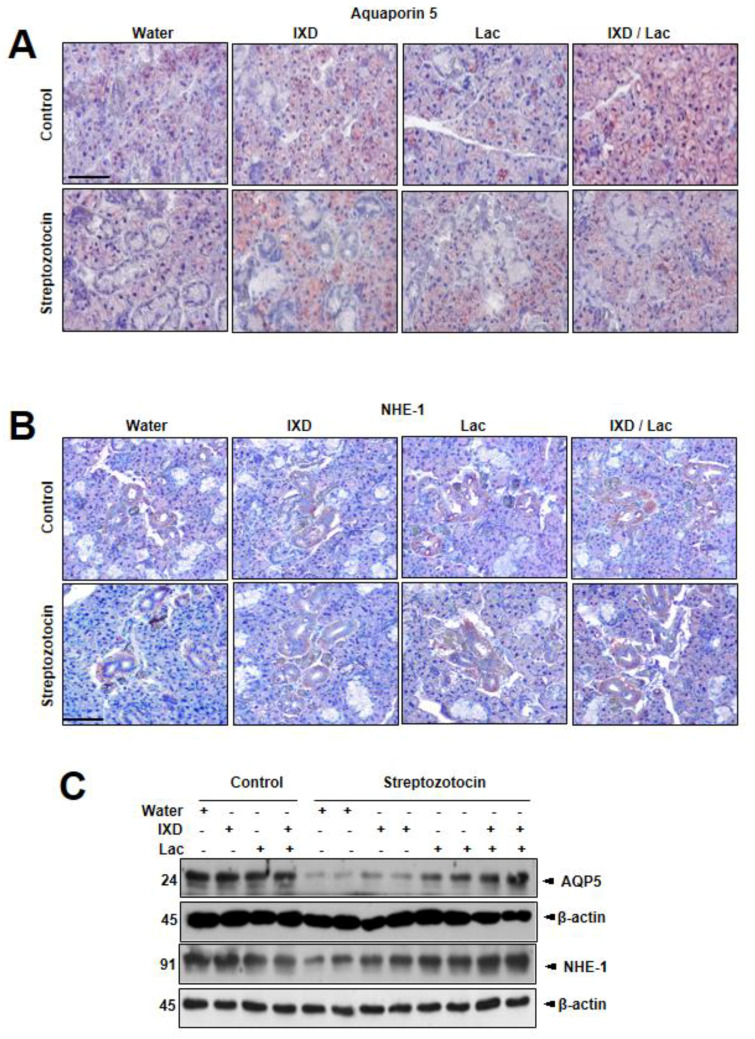
*Ixeris dentata* and lactobacillus extracts recover the disrupted aquaporin5 and sodium hydrogen exchanger1 expressions in the salivary gland of diabetic rats. Water, IXD, or lactobacillus extract was given as a spray or IXD, and subsequently, lactobacillus was given as a spray to streptozotocin-induced diabetes models. Immunohistochemistry (**A,B**) and immunoblotting (**C**) were performed with anti-aquaporin-5 or NHE-1 antibody. Magnification = 20×, scale bar = 100 μm. AQP5: aquaporin5, NHE-1: sodium hydrogen exchanger1.

Multiple reports have shown that several locations of submandibular glands expressed aquaporin 5 (AQP5) and sodium-hydrogen exchanger (NHE-1). AQP5, the main water channel to control water secretion, can be localized in the apical, basal, and lateral membranes of submandibular gland acinar cells in SD rats [23]. Localization of NHE-1 to acinar and duct cells was also reported, suggesting that the Na^+^/H^+^ antiporter isoform 1 contributed to saliva secretion [24]. In this study, AQP5 expression revealed a uniform distribution in control rats (Figure 3A), whereas diabetic rats showed weak expression in both the ductal and acinar cells of the submandibular glands. Similarly, a faint expression of NHE-1 in both the submandibular glands duct and acinar cells of diabetic rats (Figure 3B) is observed. As expected, IXD and cotreated lactobacillus extract-treated rats showed higher expression of NHE-1 in both the ductal and acinar cells of the submandibular glands compared with each treated rat. These results suggest that the cotreated IXD/lactobacillus extract increases the expression of AQP5, which controls water balance in saliva environment, i.e., submandibular acinar cells, and that the extract enhances the expression of NHE-1, which contributes to fluid secretion from acinar cells and of NaCl by duct cells. In immunoblotting data, diabetic rats had significantly lower expressions of AQP5 and NHE-1 compared with control rats (Figure 3C). Cotreated spray of IXD and lactobacillus extract more significantly recovered the reduced expressions of AQP5 and NHE-1 in diabetic submandibular glands tissue homogenates compared with either IXD or lactobacillus extract.

### 3.4. IXD and Cotreated with Lactobacillus Regulates Diabetes-Associated ER Stress

**Figure 4:**
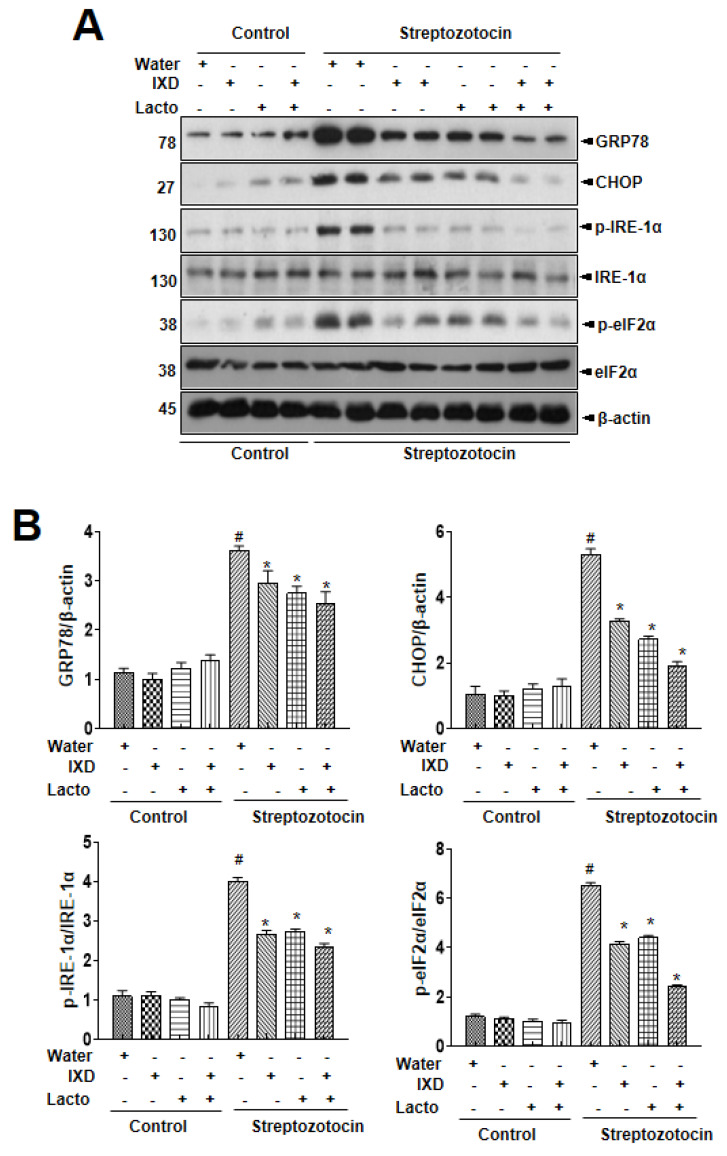
*Ixeris dentata* and lactobacillus extracts regulate ER stress response in salivary glands from diabetic rats. Water, IXD, or lactobacillus extract was given as a spray or IXD, and subsequently, lactobacillus pwas given as a spray to streptozotocin-induced diabetes models. (**A**) Immunoblotting was performed with anti-GRP78, CHOP, p-eIF2α, total eIF2α, p-IRE1α, and total IRE1α antibodies. β-actin was used as a loading control. (**B**) Bands were quantified by densitometry and normalized.

Further, submandibular gland tissue lysate was used to analyze the expression of multiple ER stress markers to assess the influence of IXD/lactobacillus on ER stress through immunoblot. It was observed that the expressions of ER stress response proteins were upregulated in diabetic rats (Figure 4). Also, no differences in the ER stress response proteins in vehicle and IXD-treated control rats were noticed. In the diabetes condition, the cotreated IXD/lactobacillus significantly inhibited the ER stress protein expression, although each single treated IXD or lactobacillus also controlled the expression, suggesting that the IXD cotreated with lactobacillus extract reduced ER stress in the diabetic submandibular gland.

### 3.5. IXD and Cotreated with Lactobacillus Protects against Streptozotocin-Induced Diabetes Model

**Figure 5:**
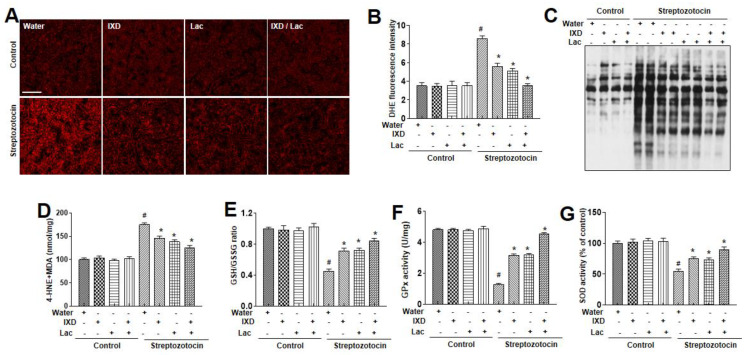
*Ixeris dentata* and lactobacillus extracts reduce lipid peroxidation and reactive oxygen species (ROS) production in salivary glands of diabetic rats. Water, IXD, or lactobacillus extract was given as a spray or IXD, and subsequently, lactobacillus was given as a spray to STZ-induced diabetes models. In the submandibular gland tissues, dihydroethidium staining was performed and quantified as described in Materials and Methods (**A, B**). On lysates from submandibular gland tissues, OxyBlot (**C**), 4-HNE and malondialdehyde analysis (**D**) were performed. The GSH/GSSG ratios (**E**), glutathione peroxidase (**F**), or superoxide dismutase (**G**) were analyzed in the submandibular gland tissues. ^#^ significant difference vs. vehicle-treated control rats; * significant difference vs. STZ-induced diabetic control rats (*p* < 0.05). Values are represented as mean ± SEM (*n* = 10 rats per group). STZ: streptozotocin, GPx: glutathione peroxidase.

Several investigations have proposed that oxidative stress significantly increases with diabetes [25,26]. Thus, to assess the oxidative stress, dihydroethidium (DHE) fluorescent staining was done to detect ROS accumulation in the submandibular gland. As shown in Figure 5A, we observed high DHE fluorescence in the submandibular glands of diabetic rats, and IXD and cotreated lactobacillus extract reduced the ROS fluorescence intensity. There were no differences in control rats treated with either water, IXD, or lactobaci3llus extract. We analyzed protein oxidation, membrane lipid peroxidation, glutathione redox status, glutathione peroxidase (GPx), and superoxide dismutase (SOD) activity in the diabetes-induced dry mouth models. The increase in protein oxidation observed in the dry mouth models was significantly reduced by the IXD combined with lactobacillus extract (Figure 5B). Since protein oxidation may be linked to ROS [27], we assessed malondialdehyde (MDA) assays, the GSH/GSSG ratio, GPx, and SOD activity. The MDA levels were reduced in the presence of IXD combined with lactobacillus extract (Figure 5C). The GSH:GSSG ratio, GPx, and SOD activities were also decreased in the diabetes models and were restored by the IXD combined with lactobacillus extract (Figure 5D–F).

## 4. Discussion

In this study, the efficacy of co-treated IXD and lactobacillus extract against diabetes-induced dry mouth was evaluated. Observations suggest that IXD combined with lactobacillus extracts exhibited a more enhancing effect on salivary secretion compared with a single treatment of either IXD or lactobacillus extract. The effect was also reflected by amylase, aquaporin 5, NHE-1 activities, and its anti-oxidative effect controlling reactive oxygen species, suggesting that the IXD/lactobacillus extract might contribute to dry mouth.

The cotreated IXD/lactobacillus showed a controlling effect against diabetes-associated dry mouth. In this study, the selection of lactobacillus and its optimized doses were based upon screening analysis of oral biofilm formation, a representative model for dental caries. Although the oral biofilm formulation is not directly related to the saliva secretion, it can, however, be considered as one of the oral health markers to ultimately contribute to saliva secretion [28,29]. Based on the test for biofilm suppression efficacy against *S. mutans*, the causative strain of tooth decay (data not shown), we selected the optimal dose of *Lactobacillus gasseri.* at “5 mg/mL” as a co-treated material with IXD extract, 50 µL [18].

In our study, submandibular gland weight and morphology were not different in diabetic rats with or without the cotreated IXD/lactobacillus; however, it did show a recovery in salivary secretion in diabetic rats with the cotreated IXD/lactobacillus (Figure 1). Specifically, in diabetic conditions, it was reported that reduction in salivary flow contributed to symptomatic drying of the oral tissues and loss of the protective effects of salivary buffers, proteins, and mucins [30]. Along with the salivary flow rate, salivary α-amylase is also one of the essential enzymes in saliva, which is an indirect saliva function marker [31,32]. In this study, the expression of α-amylase was also significantly recovered under the cotreated IXD/lactobacillus, suggesting the possibility of IXD and its compounds as a potential candidate against dry mouth [33]. Furthermore, probiotics, including lactobacillus, have been reported to have a regulatory effect on the oral functional state, such as hygiene and anti-inflammation [34,35]. The single treatment of IXD and its compounds also enhanced the saliva flow rate and amylase secretion in diabetic conditions (Figure 1, Figure 2) [18]. Through showing the more enhancing efficacy of the cotreated IXD/lactobacillus compared with the single treatment, the co-treatment is considered as a potential therapeutic/preventive approach to control dry mouth.

ER stress, a key signal to explain the disturbed folding/secretion process, was also more significantly regulated in the cotreated condition compared with the single treated condition. The ER secretory capacity is overwhelmed, causing alterations in ER folding and secretion along with an ER redox uncoupling phenomenon leading to ROS accumulation [20]. The ER stress and its associated ER-ROS accumulation contribute to hyperglycemia-associated salivary gland dysfunction and irreversible salivary gland cell damage under chronically high concentrations of glucose [21]. The ER stress and its related ROS accumulation have also been studied in IXD-treated diabetes rats [6]. In this study, the ER stress and ROS accumulation were controlled with the cotreated IXD/lactobacillus (Figure 4, Figure 5), restoring the diminished saliva flow rate and amylase secretion. Also, ROS has been proposed as a mechanism of salivary gland hypofunction in Sjogren’s syndrome [36].

Beta-adrenergic agonist-induced amylase release was suppressed by an increase in intracellular ROS and by a speedy decline in glutathione in rat parotid acinar cells, indicating the oxidative stress in salivary gland tissue. This effect brings up modifications in the secretory function and reduces salivary proteins [37].

Treatment with IXD combined with lactobacillus extract more significantly activated antioxidant enzymes, like superoxide dismutase and reduced malondialdehyde, in the dry mouth conditions compared with each single treatment (Figure 5D–H), indicating that IXD shows additive antioxidant effects when combined with lactobacillus extract. Polyphenols are a group of metabolites found in plants, but their ability of free radical scavenging makes them compound interest in plants [38,39], supporting the antioxidant activity of IXD extract that is rich in phenolic content. Specifically, the high relative contents of luteolin 7-O-glucoside and luteolin 7-O-glucuronide combined with the lactobacillus’ antioxidative effect [40] are suggested to contribute to the ROS scavenging effect in the IXD/lactobacillus-cotreated condition. The regulatory effect of ER stress and its related or unrelated ROS explains the controlling effect of IXD/lactobacillus against dry mouth, a potential mechanism in this study. Moreover, in other conditions such as aging, anti-oxidant, astaxanthin (AX) decreased the oxidative stress of serum and saliva [41]. In addition, AX controlled the inflammations in the salivary glands and increased salivary flow rate. Therefore, the antioxidant therapies, including the IXD/lactobacillus or the other anti-oxidant agents, can be expected to prevent the hyposalivation caused by oxidative stress.

Primarily, this study is designed to enhance saliva secretion without a systemic effect on the serum glucose level. The previous study has uncovered the systemic effect of an oral formula of IXD on blood glucose level, also showing the significant controlling effect against dry mouth [6]. In this study, an oral spray of IXD with lactobacillus was demonstrated to be effective against dry mouth under diabetic conditions, suggesting that the local application of the extracts might be successful in general dry mouth condition. The spray-based materials would be applicable to diabetes mellitus, type II as well as type I, because of the characteristics of the application methodology; oral spray shows a specifically local effect, not systemic effects such as glucose level changes.

In conclusion, the present study indicates that the IXD and lactobacillus extract treat or prevent diabetes-associated dry mouth, justifying their use as a functional food-originated spray formulation. The cotreated extracts exhibited the ER stress regulatory effect with the related or unrelated free radical scavenging properties. This effect was helpful in controlling dry mouth without systemic effect on blood glucose concentration in diabetic conditions.

## Supplementary Materials

The following are available online at https://www.mdpi.com/2072-6643/12/5/1331/s1, Figure S1: Schematic experimental design.
