# Supplementary figures and images for: Ixeris dentata and Lactobacillus gasseri Extracts Improve Salivary Secretion Capability in Diabetes-Associated Dry Mouth Rat Model
Source: Nutrients. 2020 May 7;12(5):1331. doi: 10.3390/nu12051331 (PMC7284355; doi:10.3390/nu12051331)

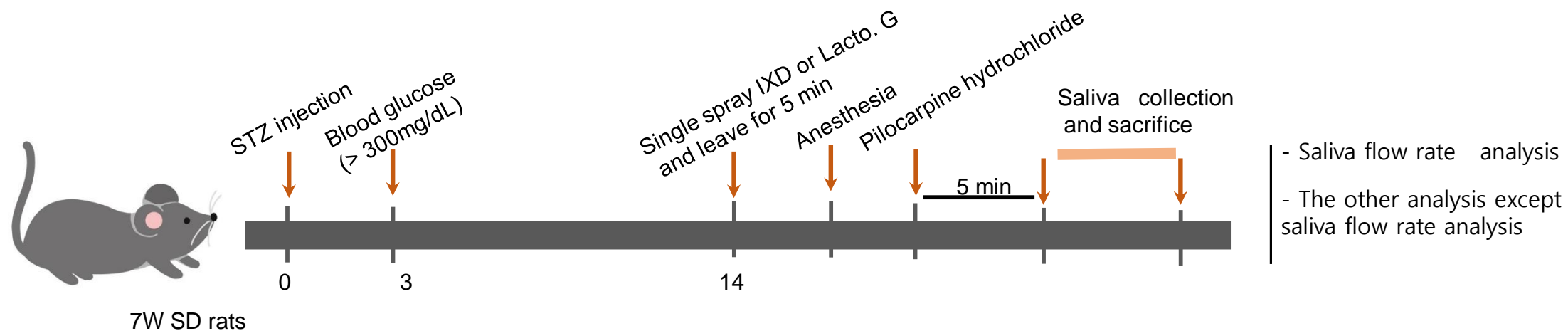

**Supplementary Figure. Schematic experimental design**

Supplement: Supplementary file 1 [file nutrients-12-01331-s001.pdf]
